# Exploring the genetic makeup and population structure among *Capsicum* accessions for crop improvement and breeding curriculum insights

**DOI:** 10.1186/s43141-022-00398-1

**Published:** 2022-08-06

**Authors:** Shamshadul Haq, Shikha Dubey, Prerna Dhingra, Kumar Sambhav Verma, Deepa Kumari, S. L. Kothari, Sumita Kachhwaha

**Affiliations:** 1 Department of Botany, University of Rajasthan, Jaipur, 302004 India; 2 Department of Genetics and Plant Breeding, UAS Dharwad, Dharwad, Karnataka 580005 India; 3 Institute of biotechnology, Amity University, Jaipur-Campus, Jaipur, Rajasthan 302006 India

**Keywords:** *Capsicum* accessions, Inter-Simple Sequence Repeats (ISSR), DNA finger printing, Genetic diversity

## Abstract

**Background:**

*Capsicum* or chilli is an important crop in India which exhibits immense structural and genetic variations reflecting their intra- and inter-specific relationships. The aim of this study was to establish relationships amongst 54 *Capsicum* accessions through analysis of genetic and population structure using ISSR markers.

**Results:**

Out of 19, successful DNA amplifications were shown by 7 ISSR primers and a total of 80 bands were identified ranging between 8 and 14 with an average of 11.43 bands/primer. A significant degree of polymorphic information content (PIC), discriminating power (DP), resolving power (RP), effective multiplex ratio (EMR), and marker index (MI) were identified as 0.39, 0.70, 6.40, 5.88, and 2.30, respectively, using ISSR markers in chillies. The cross-transferability ranged from 8.0 to 72.15% with an average of 52.63% among chillies. Amongst genetic information, grand mean values were 0.264, 0.180, 0.376, 0.296, and 0.180, which correspond to Shannon’s information index (*I*), expected heterozygosity (*He*), *Nei*’*s* gene diversity, total diversity among species (*Ht*), diversity within species (*Hs*), respectively. Further, the coefficients of gene differentiation (*Gst*) and gene flow (*Nm*) were 0.393 and 0.773, representing higher genetic variation among the population which was confirmed by analysis of molecular variance (AMOVA).

**Conclusion:**

ISSR markers represented a potent system for the estimation of relationships or variation studies and generated information useful for planning crop management and improvement strategies in chilli breeding.

## Background

Chilli or hot pepper is an important vegetable spice crop with widespread cultivation in the tropical and subtropical areas globally. The *Capsicum* genus represents a wide genetic diversity comprising 38 species [1] out of which, *C. annumm*, *C. frutescens*, *C. baccatum*, *C. chinense*, and *C. pubescens* are domesticated species worldwide [2]. Among these, *C. annumm* is a largely cultivated species, used as vegetable and spice globally. Regarding to nutritive value, chilli is a rich source of many essential vitamins, minerals, and nutrients that have a great importance for human health and consumption [3]. Besides this, chilli finds its use in pharmaceuticals and cosmetics, as natural coloring additive and in defense repellents [4]. In *Solanaceae* family, chilli harbors most complex and largest plant genome sizes, varying from 3.3 to 3.6 GB and usually with chromosome numbers 2*n* = 24 [5, 6]. Repetitive DNA elements are frequently found in its genome and constitute above 80% of the genome [4, 7]. Subsequently, it is envisaged that transposable elements are driving evolutionary forces often causing species diversification and rearrangement in chilli genomes [8]. In addition, the development of new genes by gene duplication are important for the generation of functional diversity between the species and selection of superior ones for further crop improvement or breeding processes [9, 10].

*Capsicum* species also exhibits a huge variation in morphological features, biochemical properties, and at molecular level; thus, these differences make divergences amongst species [11–13]. Also, the immense genetic diversity displayed by *Capsicum* species is an important factor that provides the information about conservation of genetic resource, breeding practices, evolutionary transitions, adaptation under biotic and abiotic pressures, and ecology and environmental relationships [14, 15]. This diversity unveils the level of delineation within or between species or populations, and these variations are very important to identify the connection between species or cultivars which apprise us about the kind of crop evolution that took place and is very supportive in the breeding programmes. Proper assessment and pattern of genetic diversity in plants or corps are invaluable for knowing the genetic variability within or across the cultivar, development of segregating progenies with maximum genetic variability from the analysis of parental combinations for further selection, and transfer of desirable genetic information from diverge germplasm into existing genetic design [16]. Hence, the assessment of genetic diversity is the key step which aid in the practices of crop improvement and breeding practices for the development of superior cultivars [17].

The last few decades have witnessed the utility of molecular marker technologies especially DNA-based marker systems in various genetics studies mainly due to their ease, quickness, and economic feasibility along with their well discriminatory potential within and across species or varieties [18]. Simultaneously, the introduction of new principles has strengthened a molecular marker technology for sophisticated exploration of genetic variation analysis that have provided simple and easy platform for determining morphological, ecological, conservatory, and evolutionary relationship within and across species [19, 20]. Molecular markers are more decisive and preferable for identifying genetic variation because they are inert to environmental pressure and have the capacity to distinguish a variation at genome level making them more suitable to assess genetic diversity [3]. At present, frequently used DNA-based markers or molecular markers are restriction fragment-length polymorphisms (RFLPs), random amplified polymorphic DNA (RAPDs), amplified fragment-length polymorphisms (AFLPs), inter-simple sequence repeats (ISSRs), simple sequence repeats (SSRs), single-nucleotide polymorphisms (SNPs), start codon-targeted polymorphism (SCoT), etc. [21]. Using these marker technologies, numerous studies have been conducted in different plant species depending on specific genetic applications desired by various research groups [3, 22–26].

In the present study, assessment of genetic variation was done using ISSR markers to sketch a comparative overview of degree of genetic polymorphisms, primer efficiency, cross-transferability, and genetic and structural plasticity among 54 *Capsicum* accessions. ISSR marker system offers quick, easy handling, reliable, cost-effective, and highly informative method for a variety of genetic applications [26]. ISSR markers are highly reproducible that target microsatellites which are densely distributed throughout the plant or eukaryotic genome and reveal increased level of polymorphisms due to their higher annealing temperature and longer primer sequence length along with no requirement of prior information of flanking sequence like SSRs [27]. The advantages with this marker system comprise that they are present in both nuclear and organelle genomes, and their segregation follows the Mendelian rule as dominant markers and are highly polymorphic [28, 29]. Also, ISSR markers have proven their supremacy in variety of applications such as cultivar identification, genetic diversity, gene tagging, genome mapping, molecular ecology, phylogenetic studies, plant breeding, and evolutionary analysis [30–34].

## Materials and methods

### Plant materials and growing conditions

A set of 54 accessions of *Capsicum* was procured from various research centres in India namely: Agriculture Research Station, Jodhpur; Indian Institute of Vegetable Research, Varanasi (ICAR-IIVR); School of Life Science, Jawaharlal University, New Delhi; National Bureau of Plant Genetic Resources, Hyderabad; and National Seeds Corporation, Hyderabad. These chilli accessions comprised 49 varieties of *C. annumm*, 3 varieties of *C. baccatum*, and 2 varieties of *C. frutescens* (Table 1). Seeds were planted in a seed tray and kept in a plant growth chamber under controlled growth environments 26 ± 1°C temperature, 16 h photoperiod, and 300 μmol/m^2^ s^-1^ photosynthetic photon fluxes according to the method explained by Gupta [3].

**Table 1:** *Capsicum* accessions used for the genetic assessment study

Sr. No.	Species	Accessions	Sr. No.	Species	Accessions
1	*C. annuum*	EC-596878	28	*C. annuum*	Panjab Lal up
2	*C. annuum*	EC-596920	29	*C. annuum*	Pant C-1 up
3	*C. annuum*	EC-596940	30	*C. annuum*	Arka Abhir
4	*C. annuum*	EC-599955	31	*C. annuum*	Kashi Anmol
5	*C. annuum*	EC-599977	32	*C. annuum*	Jayanti
6	*C. annuum*	IC-328725	33	*C. annuum*	LCA-423
7	*C. annuum*	IC-361989	34	*C. annuum*	LCA-402
8	*C. annuum*	IC-372043	35	*C. annuum*	EC-391075
9	*C. annuum*	IC-565081	36	*C. annuum*	LCA-440
10	*C. annuum*	IC-572470	37	*C. annuum*	LCA-443
11	*C. annuum*	IC-572481	38	*C. annuum*	LCA-434
12	*C. annuum*	Pusa Sada Bahar	39	*C. annuum*	LCA-422
13	*C. annuum*	Pusa Jwala	40	*C. annuum*	LCA-403
14	*C. annuum*	Pant Chilli-1	41	*C. annuum*	LCA-353
15	*C. annuum*	Chilli G-4	42	*C. annuum*	LCA-335
16	*C. annuum*	Chilli G-5	43	*C. annuum*	LCA-427
17	*C. annuum*	GKC29	44	*C. annuum*	LCA-235
18	*C. annuum*	Punjab Lal	45	*C. annuum*	LCA-206
19	*C. baccatum*	EC-382035	46	*C. annuum*	LCA-334
20	*C. baccatum*	IC-315759	47	*C. annuum*	AKC-89/38UP
21	*C. baccatum*	PBC-81	48	*C. annuum*	EC-341094
22	*C. frutescens*	NMCA-40008	49	*C. annuum*	EC-518968
23	*C. frutescens*	COO-309	50	*C. annuum*	EC-566320
24	*C. annuum*	Phule Jyoti	51	*C. annuum*	EC-622085
25	*C. annuum*	Byadigi Kaddi	52	*C. annuum*	EC-596958
26	*C. annuum*	Byadigi Dabbi	53	*C. annuum*	EC-497632
27	*C. annuum*	Kashi Gaurav	54	*C. annuum*	NIC-268216

### DNA extraction and purification

DNA extraction was carried out from fresh young leaves (5g) using CTAB method (Doyle & Doyle, 1990) with minor modifications. The leaves were grinded in extraction buffer [1 M Tris (pH 8.0), 0.5 M EDTA, 5 M NaCl, and 200 μM β-mercaptoethanol] and incubated at 65°C for 1 h followed by chloroform:isoamyl alcohol (24:1, v/v) treatment. DNA pellet obtained in chilled isopropanol was washed with 70% ethanol. Isolated DNA samples were treated with RNase for 1 h at 37°C followed by phenol:chloroform:isoamyl alcohol (25:24:1 v/v/v) treatment then washed with 70% ethanol. DNA pellet was air-dried and dissolved in TE buffer and then stored at −80°C [35]. The quality of DNA was checked on a 0.8% agarose gel stained with ethidium bromide, and DNA concentration was adjusted to 25 ng/μL using known concentration of λ DNA for each polymerase chain reaction (PCR).

### ISSR-PCR and electrophoresis

A total of 19 ISSR primers (University of British Columbia, primer set no. 9, Vancouver, Canada) were examined for DNA amplifications in chilli accessions. Finally, 7 ISSR primers were selected for analysis among 54 chilli accessions due to their sharp and clear banding profiles. All PCR reactions were performed in the final volume of 10 μl each using thermal cycler (BioRad, UK). Each reaction mixture contained 1 μl of DNA template (25 ng), 1.0 μl Taq buffer (10X) with 2.5 mM of MgCl_2_, 1 μl of primer (10 pmole/ μL), 0.25 μl of dNTPs (100 mM), and 0.1 μl of Taq DNA polymerase (0.5 U). PCR amplification conditions included initial denaturation at 94°C for 3 min followed by 35 cycles which included denaturation at 94°C for 1 min followed by annealing at 45 to 51°C for 1 min depending upon primers and then extension at 72°C for 2 min with final extension at 72°C for 7 min. All amplified products were separated through agarose gel electrophoresis using 1.2% agarose gel (Himedia) in 0.5× TBE (Tris-Borate- EDTA) buffer for ∼1.5 h at 70 V. Gel was stained with ethidium bromide dye, and BioRad gel doc system was used for visualization of DNA bands and further analysis.

### Estimation of ISSR marker efficiency

Clear and reproducible amplified bands obtained from the DNA amplifications profile for each ISSR primer were used for the experiment and scored as binary matrix, 1 for the presence and 0 for the absence. The efficiency of ISSR markers was calculated as described by polymorphic information content (PIC), discriminating power (DP), resolving power (RP), effective multiplex ratio (EMR), and marker index (MI) using iMEC platform [36]. The relative primer polymorphisms and cross-transferability were measured within and across different chilli accessions using ISSR markers [25].

### Genetic and structural measurement in the population and statistical analysis

Parameters such as the number of different allele (*Na*), number of effective allele (*Ne*), Shannon’s information index (*I*), expected heterozygosity (*He*), unbiased expected heterozygosity (*uHe*), analysis of molecular variance (AMOVA), and principal coordinate analysis (PCoA) were evaluated through GenALEx 6.5 program [37]. Further, the factors namely Nei gene diversity, total species diversity (*Ht*), diversity within population (*Hs*), coefficient of gene differentiation (*Gst*), and gene flow (*Nm*) were examined to evaluate genetic flow using the POPGENE 1.32 software [38]. The genetic similarity among different chilli accession was identified by FreeTree software which generated a similarity matrix based on Jaccard’s similarity coefficient [39], and this matrix was further utilized to generate dendrogram based on UPGMA (Unweighted Pair Group Method Using Arithmetic Averages) algorithm using TreeView X software [40].

Structural plasticity in the different chilli accessions was further evaluated by Euclidean similarity index and correlation matrix, and both were characterized according to Fruchterman-Reingold algorithm which computes the biological data according to space filling curve manner [41] using PAST 4.02 statistical software [42]. In order to confirm subpopulation (*K*) numbers in *Capsicum* accessions, the genetic makeup was further explored by STRUCTURE software version 2.3.4 based on Bayesian model-based clustering analysis [43]. To identify putative subpopulation (*K*), each chilli accession was tested for *K* = 1 to *K* = 10 with admixture model and correlated allele frequencies. The five independent runs were assessed for each fixed *K* with a burn-in period of 10,000 and 100,000 Markov chain Monte Carlo (MCMC) iterations. The optimum *K* value was examined by *ΔK* statistic and L (*K*) [44] using Structure Harvester program [45].

## Results

### ISSR markers and GC content

Initially, 19 ISSR markers were used for the analysis amongst which 7 ISSR markers were retained due to their successful amplification amongst different *Capsicum* accessions. The GC-content of the 7 selected primers were belonged to 47% and 53% whereas 4 primers revealed 53% GC content and other 3 primers displayed 47% GC content. All the selected primers anchored with different dinucleotide repeat microsatellites with each having 17 bp long sequence, and their annealing temperatures ranged from 45 to 51°C (Table 2).

**Table 2:** Depiction of primers used in the study and their amplification efficacy

S.N.	ISSR primers	Sequence (5’ – 3’)	GC-content	Annealing temperature	Total bands	Range (bp)	Polymorphism information content (PIC)	Discriminating power (DP)	Resolving power (RP)	Effective multiplex ratio (EMR)	Marker index (MI)
1	UBC 807	(AG)_8_T	47	51°C	12	153-883	0.407	0.527	5.778	8.26	3.36
2	UBC 808	(AG)_8_C	53	51°C	14	187-941	0.401	0.891	7.704	4.63	1.85
3	UBC 809	(AG)_8_G	53	51°C	13	168-1499	0.375	0.780	7.926	5.63	2.11
4	UBC 810	(GA)_8_T	47	45°C	10	225-1434	0.414	0.495	5.185	7.11	2.95
5	UBC 811	(GA)_8_C	53	45°C	11	141-892	0.376	0.704	6.222	5.44	2.04
6	UBC 813	(CT)_8_T	47	49°C	12	324-2265	0.374	0.779	7.148	5.65	2.12
7	UBC 818	(CA)_8_G	53	45°C	8	162-1238	0.376	0.694	4.852	4.43	1.67
**Average**			**50.43**	**48.14**	**11.42**	**583.38**	**0.389±0.017**	**0.696±0.142**	**6.402±1.216**	**5.88±1.36**	**2.30±0.62**

### ISSR-PCR amplification in Capsicum accessions

**Fig. 1:**
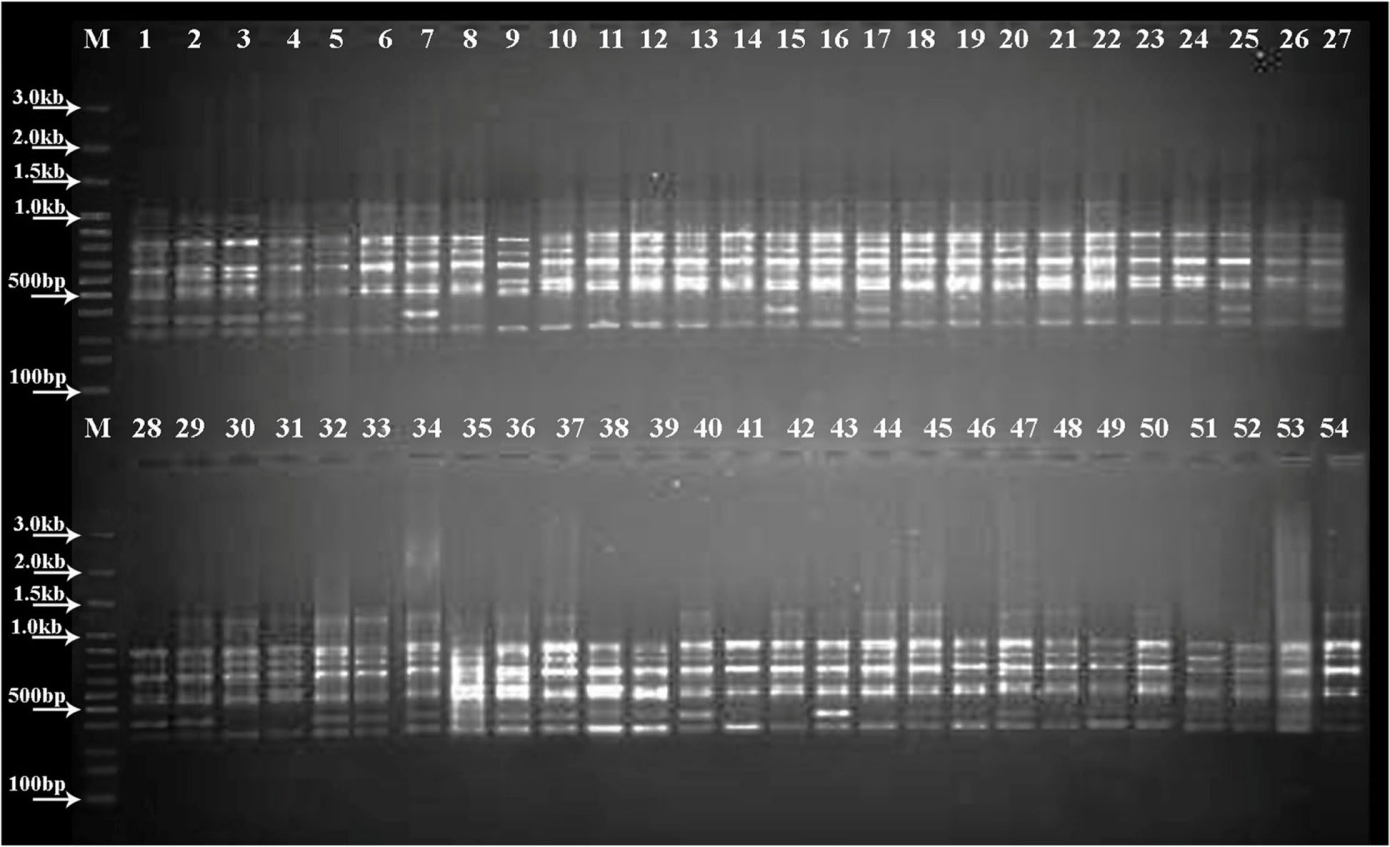
Amplification profile of UBC 807 primer among 54 *Capsicum* accessions. In the figure, M represents the DNA ladder and Lanes 1–27 and 28–54 represent different *Capsicum* accessions which are displayed in Table 1

Out of 19 primers, 7 primers showed a successful DNA amplification at different annealing temperature and rest of them were unable to retain any PCR amplification amongst different *Capsicum* accessions. A total of 80 DNA amplicons or bands were obtained ranging from 8 to 14 bands with an average of 11.42 bands per primer. Amongst *Capsicum* accessions, the maximum banding patterns were observed in *C. annumm* followed by *C. baccatum* and *C. frutescens* (Fig 1). The primer UBC 808 (14 bands) and UBC 818 (8 bands) showed increased and reduced DNA banding profile, respectively, and the size of DNA amplicons ranged from 141.15 to 2265.32 bp with an average of 583.38 bp in size (Table 2).

### Efficiency of ISSR marker in Capsicum accessions

The efficiency of ISSR markers amongst different *Capsicum* accessions was identified through the estimation of various parameters such as polymorphic information content (PIC), discriminating power (DP), resolving power (RP), effective multiplex ratio (EMR), and marker index (MI). The PIC ranged from 0.37 (UBC 813) to 0.42 (UBC 810) with an average of 0.39. Moreover, the differential DNA banding pattern amongst chilli accessions was defined by DP and the average value was 0.7 which ranged from 0.45 (UBC 10) to 0.89 (UBC 808). Distribution of DNA banding among chilli accessions was calculated by RP which ranged from 4.9 (UBC 813) to 7.9 (UBC 809) with an average of 6.40 RP (Table 2). Further, an average EMR was 5.88 ranging from 4.43 (UBC 818) to 8.26 (UBC 807) and MI ranging from 1.67 (UBC 818) to 3.36 (UBC 807) with an average of 2.30 was observed. Thus, ISSR markers revealed significant genetic polymorphism amongst different chilli accessions taken for this study.

### Primer polymorphism and cross-transferability in Capsicum accessions

**Fig. 2:**
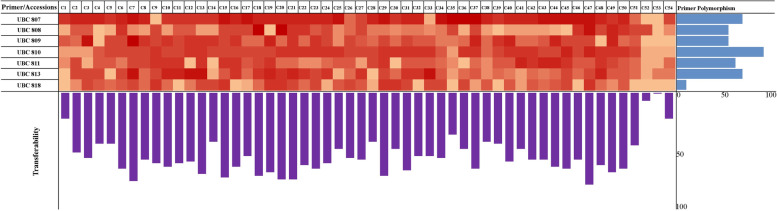
Details of the cross-amplification or cross-transferability and marker polymorphism among the 54 different *Capsicum* species. Light and dark colour boxes represent high and low level of DNA banding in the respected chilli accessions under marker or primer amplification. The bars represent the percentage of marker polymorphism and cross-transferability

Depending upon the banding profile amongst chilli accessions, the primer polymorphism falls in a range from 79.62% (UBC 818) to 100% (UBC 810) with an average of 91.80% polymorphism. Moreover, the cross-amplification potential or cross-transferability of primers was further identified amongst different chilli accessions and it ranged from 8.0 to 72.15% with an average of 52.63% (Fig. 2). Accessions EC-497632 and AKC-89/38UP showed a reduced and increased cross-transferability, respectively, and some other accessions IC-361989, Chilli G-4, and Pant C-1 UP also revealed significantly increased cross-transferability amongst *C. annumm*. However, significantly reduced cross-transferability was also identified in EC-596958, EC-596878, NIC-268216, and EC-391075 and moderate level of cross-amplification also observed in *C. annumm* accessions. All the accessions belonging to *C. baccatum* namely EC-382035, IC-315759, and PBC-81 showed improved cross-transferability. Elevated level of cross-amplification was also observed in EC-382035 and IC-315759 accession belonging to *C. frutescens*.

### Characterization of genetic structure in Capsicum species

The genetic information ascertained at ISSR marker level among various chilli accessions revealed that *Na* was common for all the ISSR markers followed by *Ne* which varied from 1.442 (UBC 7) to 1.779 (UBC 8) and *I* ranged from 0.428 (UBC 7) to 0.616 (UBC 8), while *He* ranged from 0.275 (UBC 7) to 0.428 (UBC 8) and *uHe* were in between 0.277 and 0.432 for UBC 7 and UBC 8, respectively. Nei’s gene diversity ranged from 0.283 (UBC 808) to 0.432 (UBC 807) with an average of 0.383 while *Ht* ranged from 0.231 (UBC 807) to 0.354 (UBC 818) with an average of 0.297 and an average *Hs*; 0.178, which ranged from 0.146 (UBC 807 and UBC 813) to 0.219 (UBC 808), while the average of *Gst* was 0.337 which varied from 0.228 (UBC 808) to 0.449 (UBC 818) and *Nm* varied from 0.712 (UBC 818) to 6.171 (UBC 808) with an average of 3.261 (Table 3). Hence, the grand mean values of genetic parameters; Nei gene diversity, *Ht*, *Hs*, *Gst*, and *Nm* were 0.376, 0.296, 0.180, 0.393, and 0.773, respectively (Table 4).

**Table 3:** Characterization of a genetic plasticity at a marker level among chilli accessions

Primers	***Na***	***Ne***	***I***	***He***	***uHe***	Nei gene diversity	***Ht***	***Hs***	***Gst***	Nm*
**UBC 807**	1.167±0.081	1.267±0.052	0.208±0.041	0.145±0.028	0.147±0.029	0.432±0.092	0.231±0.077	0.146±0.029	0.324±0.143	1.546±1.612
**UBC 808**	1.476±0.084	1.384±0.038	0.337±0.030	0.225±0.021	0.256±0.025	0.282±0.154	0.315±0.182	0.219±0.114	0.228±0.189	6.171±9.663
**UBC 809**	1.282±0.080	1.353±0.040	0.295±0.032	0.201±0.022	0.222±0.024	0.372±0.130	0.295±0.154	0.201±0.125	0.276±0.225	5.605±9.473
**UBC 810**	1.233±0.078	1.275±0.048	0.217±0.038	0.151±0.026	0.156±0.027	0.400±0.130	0.237±0.126	0.151±0.072	0.299±0.168	3.450±7.024
**UBC 811**	1.233±0.107	1.338±0.047	0.280±0.037	0.191±0.025	0.204±0.027	0.402±0.142	0.338±0.151	0.191±0.083	0.353±0.253	3.747±7.068
**UBC 813**	1.194±0.085	1.249±0.042	0.216±0.036	0.146±0.024	0.153±0.025	0.370±0.110	0.309±0.159	0.146±0.068	0.430±0.250	1.597±2.038
**UBC 818**	1.417±0.073	1.344±0.055	0.277±0.041	0.191±0.029	0.208±0.032	0.422±0.101	0.354±0.093	0.191±0.043	0.449±0.110	0.712±0.473
Average	1.286±0.084	1.316±0.046	0.262±0.036	0.179±0.025	0.383±0.027	0.383±0.123	0.297±0.134	0.178±0.076	0.337±0.191	3.261±5.336

*Na*, observed number of alleles; *Ne*, effective number of alleles; *I*, Shannon’s Information Index; *Ht, *total diversity; *Hs*, diversity within population; *Gst*, coefficient of gene differentiation; Nm*, gene flow

**Table 4:** Characterization of a genetic plasticity at species level among chilli accessions

Species	***Na***	***Ne***	***I***	***He***	***uHe***	Nei gene diversity	***Ht***	***Hs***	***Gst***	Nm*
*Capsicum annumm*	2.000±0.000	1.656±0.034	0.549±0.018	0.373±0.015	0.377±0.015					
*Capsicum baccatum*	1.114±0.079	1.223±0.042	0.181±0.032	0.124±0.022	0.149±0.027					
*Capsicum frutescens*	0.747±0.071	1.072±0.024	0.061±0.021	0.042±0.014	0.056±0.019					
**Grand mean**	**1.287**±**0.049**	**1.317**±**0.025**	**0.264**±**0.019**	**0.180**±**0.014**	**0.194**±**0.015**	**0.376**±**0.132**	**0.296**±**0.021**	**0.180**±**0.008**	**0.393**	**0.773**

*Na*, observed number of alleles; *Ne*, effective number of alleles; *I*, Shannon’s Information Index; *Ht*, total diversity; *Hs*, diversity within population; *Gst*, coefficient of gene differentiation; Nm*, gene flow

### Population structure of Capsicum accessions

**Fig. 3:**
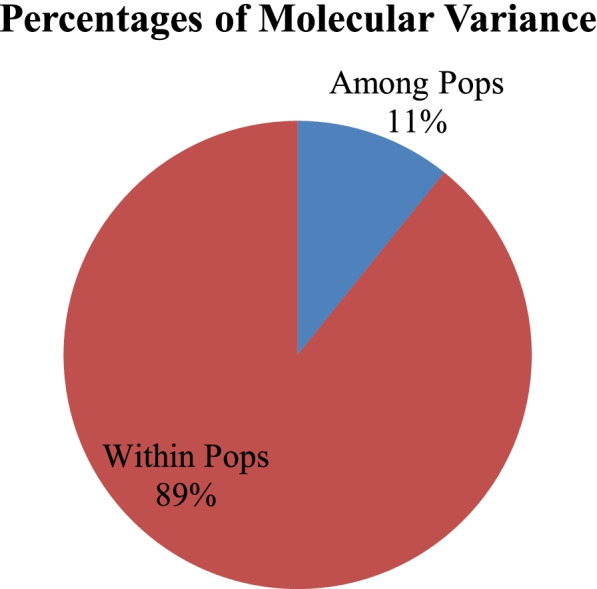
Analysis of molecular variance (AMOVA) performed among the 54 *Capsicum* accessions

**Fig. 4:**
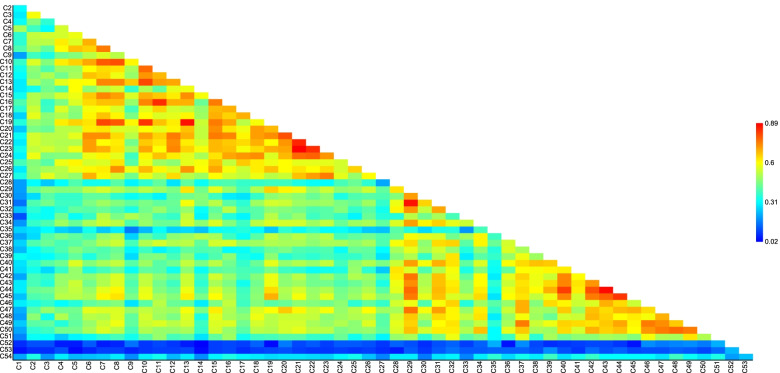
Jaccard’s similarity coefficient analysis amongst 54 different *Capsicum* accession. Red and blue colors correspond to high and low degree of similarity between the accessions

**Fig. 5:**
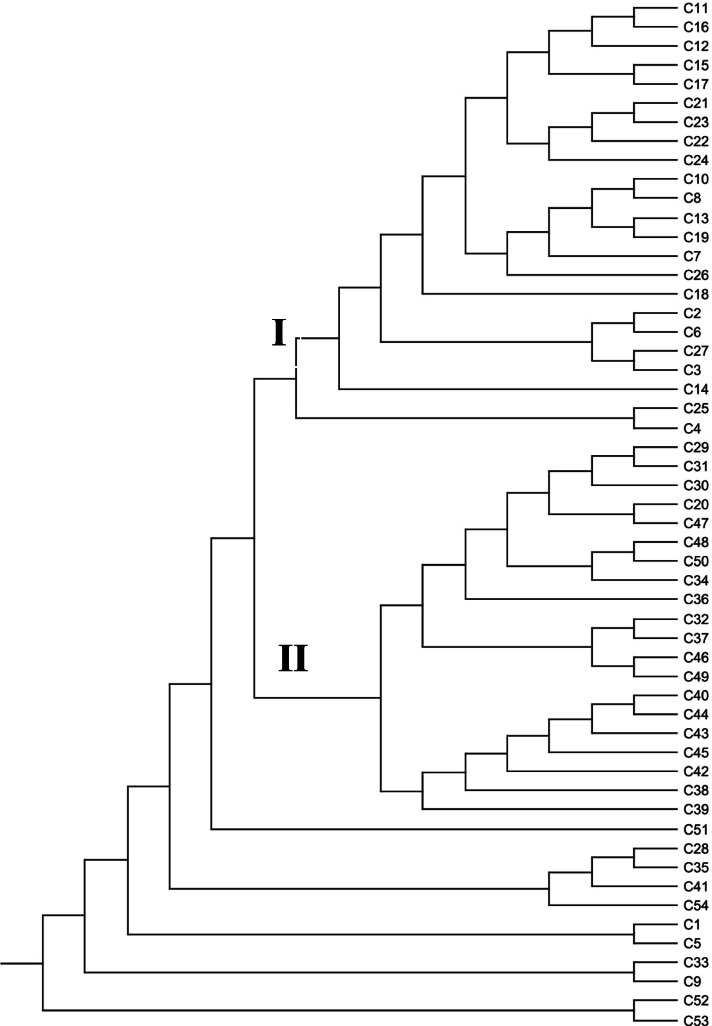
UPGMA clustering analysis based on Jaccard’s similarity coefficient among 54 different chilli accessions

A significant genetic differentiation was observed within and across chilli accessions using AMOVA (*P* < 0.001) which is useful for partitioning of the overall variation. The results indicated that 89% of total variance occurred within chillies and 11% among chillies (Fig. 3). The structural plasticity in the *Capsicum* population was further identified by Jaccard’s similarity coefficient, UPGMA clustering, Euclidean similarity, correlations, and principal coordinate analysis (PCoA) among the different *Capsicum* accessions. The Jaccard’s similarity coefficient fluctuated from 0.02 to 0.89, and maximum similarity was observed between different *Capsicum* accessions in the order: 0.89 between C23 & C21, 0.85 in C21 & C22, 0.84 in C22 & C23 and C11 & C16, 0.81 in C10 & C13 and C7 & C19, and 0.80 in between C20 & C21 and C42 & C44 (Fig. 4). The genetic relatedness analyzed amongst *Capsicum* accessions by UPGMA cluster analysis. Broadly, two major (I and II) groups were found with 18 and 13 chilli accessions which were placed into distinct branches in the dendrogram along with several other loose clusters containing few chilli accessions. It was observed that all the *C. baccatum* and *C. frutescens* accessions represented closeness and were placed in group I; on the other hand, they also showed association of several *C. annumm* accessions in the present study (Fig. 5).

**Fig. 6:**
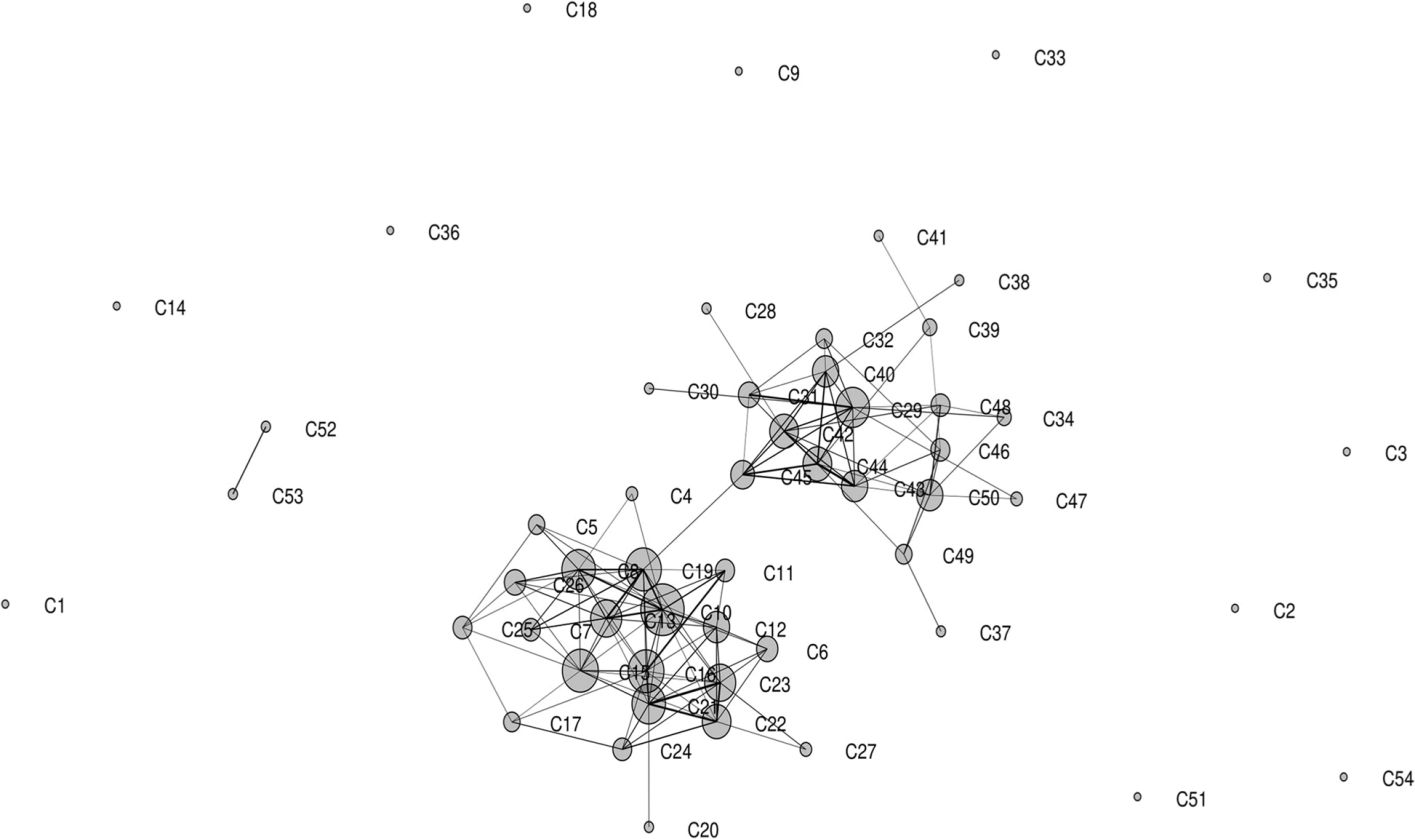
Euclidean similarity index with Fruchterman-Reingold algorithm data-based population characterization among 54 different *Capsicum* accessions

**Fig. 7:**
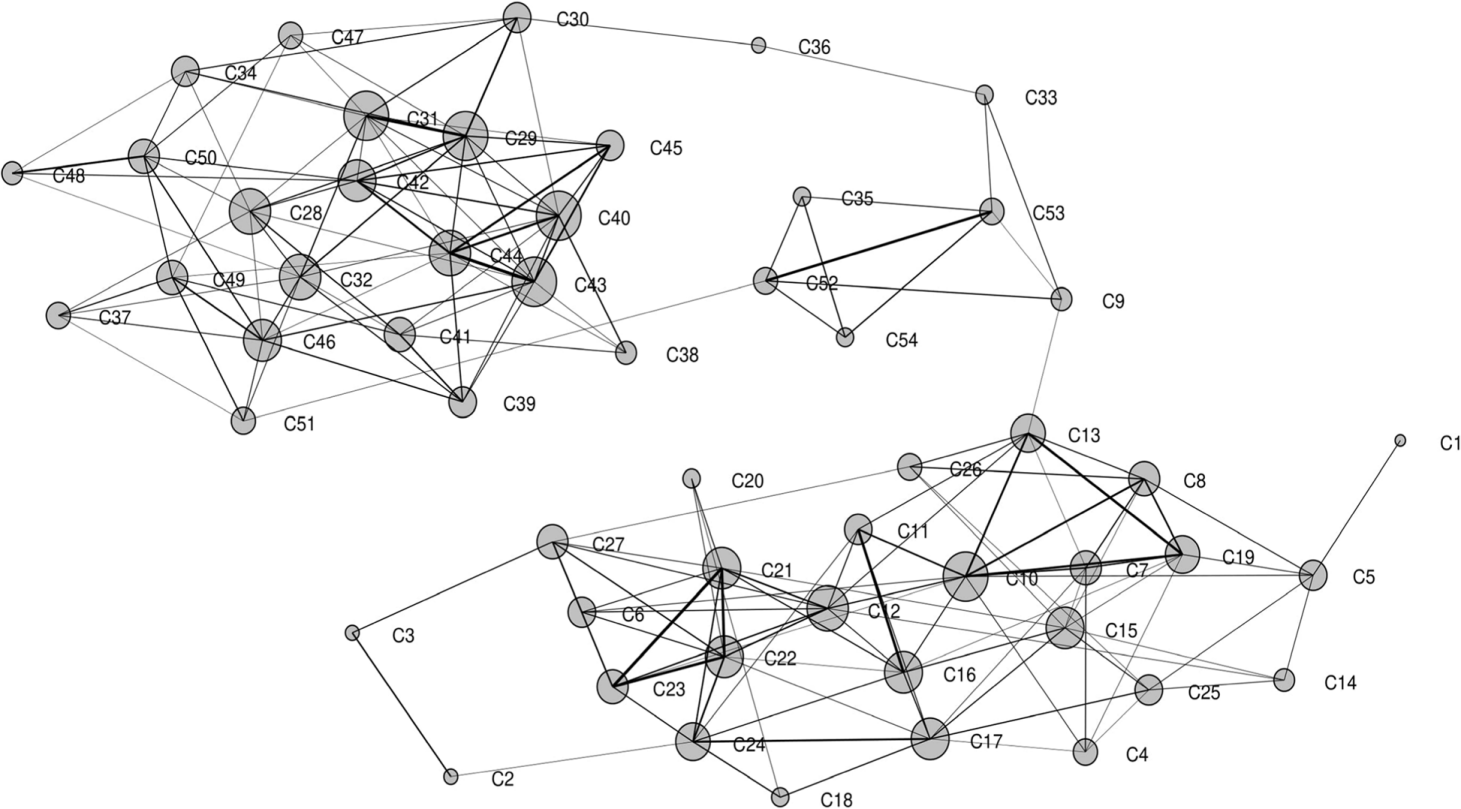
Representation of correlation analysis with Fruchterman-Reingold algorithm among 54 different *Capsicum* accessions

The two major groups along with several loose associations were also observed through Euclidean similarity index associated with Fruchterman-Reingold algorithm which explained an intuitive and efficient representation of different *Capsicum* accessions into space-filling curves manner and provided a new way for the computation of biological data (Fig. 6). Similarly, the representation of relatedness and grouping were further supported by correlation matrix associated with Fruchterman-Reingold algorithm amongst different chilli accessions (Fig. 7). Major finding of present study indicates that broadly two major set of associations with inter- and intra-locking relation within chilli accessions with some reduced networks which were revealed within chillies.

**Fig. 8:**
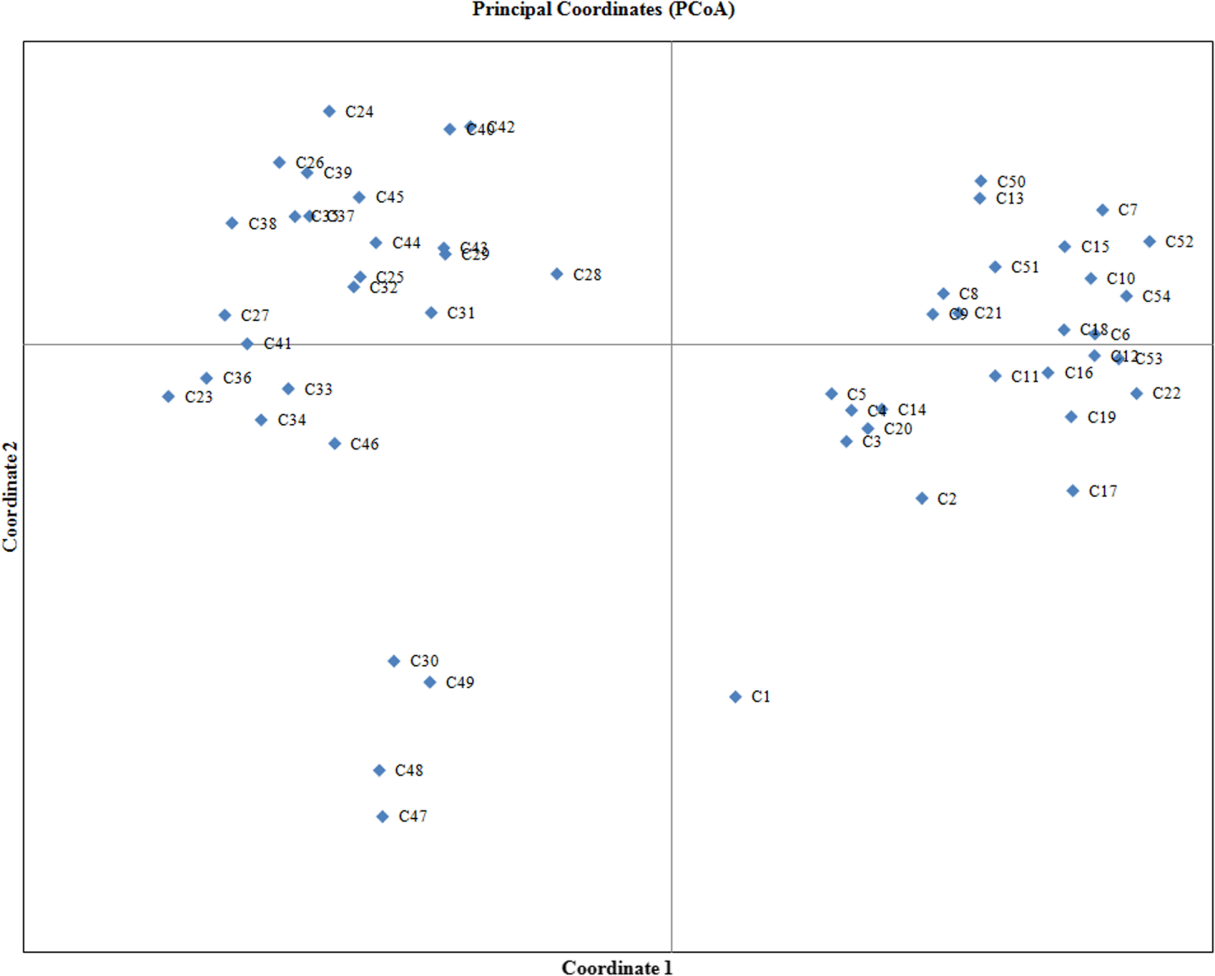
Principal coordinate analysis (PCoA) of 54 *Capsicum* accessions. Horizontal and vertical scales correspond to the first and second principal axes of variation, respectively, which represents the degree of variations among various chilli accessions

In addition, principal coordinate analysis (PCoA) was performed to visualize population structure for 54 different chilli accessions and the results of first three PCoA accounted 39.18% of total variation. Based on the PCoA outcomes, two groups were differentiated majorly with densely concentrated *Capsicum* accessions besides several other clustering which disclosed few members of *Capsicum* accessions (Fig. 8). Hence, the results of PCoA were found to be consistently similar with those accomplished by UPGMA clustering, and Euclidean similarity index and correlation matrix with Fruchterman-Reingold algorithm.

**Fig. 9:**
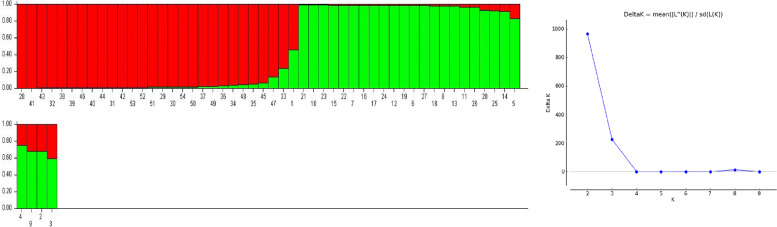
Inferred population structure of 54 *Capsicum* accessions based on ISSR markers using STRUCTURE program which observed at *K* = 2. The maximum value of *ΔK* was determined at *K*=2 by STRUCTURE HARVESTER

In order to confirm reliability of most likely grouping in 54 *Capsicum* accessions, an analysis was performed using Structure software. The maximum *ΔK* was observed at *K* = 2 with accessions falling into two groups, and the overall proportion of the samples in each of the two groups were 0.529 and 0.471 (Fig. 9). The inferred population structure for *K* = 2 showed that 89% of the accessions have a membership coefficient (qi) to one of the subpopulations higher than 0.8, while the rest could be considered as admixed (qi≤0.8). Thus, the outcome obtained from structure analysis revealed that all the accessions were categorized into two groups, which is in consistency with results retrieved from aforesaid mention in UPGMA, Euclidean similarity, correlation matrix, and PCoA results.

## Discussion

*Capsicum* is one of the most important crops in India, comprising of agro-morphologically distinct *Capsicum* varieties, and India is known to be the biggest contributor for both production and consumption of *Capsicum*. Aside from variation in growing areas, *Capsicum* fruits also displayed a variation in the size, shape, color, taste, shelf life, and chemical composition [46]. Moreover, knowledge of variation in the genome size, genetic plasticity, level of adulteration, fruit quality, pungency, size, and color is very important parameters for breeding advancement programs in chillies. For deciphering variation in *Capsicum* species, morphological indicators have played a big role, among which flower and fruit characteristics are most important [47–50], in which biochemical, physiological, and molecular aspects are also extensively investigated [3, 51–54]. Though morphological and biochemical characters are credible scores for evaluating variation in *Capsicum* species but are also subject to change under different environmental conditions [55, 56], therefore accessing genetic diversity using molecular markers is more advantageous because molecular markers are phenotypically neutral and not regulated by environmental conditions. Several workers have attempted to unveil genetic diversity in *Capsicum* species using various molecular markers, such as AFLP [57], SSRs [58], RAMPO [59], and RAPD [60], but extensive studies using ISSR markers are sparsely available [61]. ISSR markers are dominant markers comprising of polymorphic arbitory primers with high reproducibility and requiring high stringency in PCR conditions compared to RAPD markers system. Following Mendelian fashion of inheritance, this technique includes microsatellite repeats (di, tri, tetra, or penta nucleotides) unit bearing oligo-nucleotide primers, non-anchored, or anchored at the 5′ or 3′ end with 1 to 4 degenerate nucleotides and generally 16 to 25 nucleotides long [62, 63].

The efficiency of ISSR markers have been utilized in various plant such as *Solanum lycopersicum* [64], *Jatropha curcas* [23], *Cymbopogon germplasms*, [63] *Citrullus colocynthis* [26], *Arabidopsis thaliana* [65], and *Triticum durum* [66]. The aforsaid ISSR accomplishments in various plants paved the way to undertake the present study to establish the genetic correlation among 54 different *Capsicum* accessions comprising three distinct *Capsicum* species (*C. annuum*, *C. baccatum*, and *C. frutescens*). The ISSR marker technique is a well-established significant approach for exploring the varieties for useful applications such as germplasm identification, parentage inquiry, genetic diversity, gene mapping, QTL (quantitative trait loci) analysis, evolutionary strategy, and taxonomic studies [30, 67–70].

In the present study, an average GC-content of the 7 selected ISSR markers was 54.28% which is in consistent with previous reports [31, 71]. A total of 80 bands were generated from 7 ISSR primers selected in the present study, and the average frequency of banding pattern was 11.43 bands per primer, while in another study in *Capsicum*, 2 ISSR primers amplified a total 38 bands with an average of 19 bands per primer [61]. Enhanced marker efficacy indices such as PIC, DP, RP, EMR, and MI reflect the discriminatory potential of ISSR markers [36]. PIC is a measure of quality or informativeness of polymorphism which is defined by the number and frequency of the alleles generated by given molecular marker, and thus, values in between 0 and 0.5 correspond to dominant marker while in between 0 and 1 correspond to co-dominant marker [36, 72]. In the present study, PIC ranged from 0.37 to 0.42 with an average of 0.39 PIC which is in compliance with 0.40 PIC reported while evaluating genetic diversity based on fruit pericarp in *Capsicum annuum*. An average PIC value of 0.156 was reported for 237 accessions of *C. baccatum*, *C. annuum*, *C. chinense*, *and C. frutescens* using AFLP markers [57], whereas deciphering genetic diversity in chilli germplasm, average PIC was observed to be 0.69 using SSR markers and 0.63 using RAMPO markers in 48 Chilli accessions [59] and 0.77 PIC was observed using two ISSR markers in chilli accessions [61].

Likewise, the RP value corresponds to the effectiveness of the marker for identification of variation, and in the present study, an average RP of 6.40, ranging from 4.9 to 7.9, was recorded, which is similar to genetic analysis done in different plant species [26, 73–75], but contrary to this, 16.08 Rp value was recorded using two ISSR primers in 12 *Capsicum* accessions [61]. A significant polymorphism within the accession is measured by DP while lower and higher values of DP represent highly and reasonably polymorphic nature of marker between the accessions. In this study, DP ranged from 0.45 to 0.89 with an average of 0.7 DP in different chilli accessions which is in consensus with outcome of several analysis performed with ISSR makers in different plant species [36, 76, 77], whereas in chilli germplasm using 7 SSR primers an average DP value of 0.40 was observed [58]. On the basis of allelic frequency, the informativeness of makers may vary between the gene pool but the most informative remark is designated to those makers which exhibits increased DP value which corresponds to high discriminatory power in gene pools [36, 78]. Furthermore, a significant level of EMR and MI was observed which revealed the success of ISSR markers among *Capsicum* accessions. Therefore, the selected 7 ISSR markers reflected a significant genetic polymorphism and genetic information, indicating their effectiveness to differentiate various chilli accessions.

Primer polymorphism ranged from 79.62 to 100% with an average of 91.80% polymorphism amongst different chilli accessions in the present study which is close to 91.3% polymorphism as depicted in chilli accessions using ISSR markers [61]. In another study, primer polymorphism ranged from 50 to 100% with an average of 81.52% amongst various *Capsicum* accessions using SCoT markers [3]. The extent of cross-amplification or cross-transferability of ISSR maker ranged from 8.0 to 72.15% with an average of 52.63% amongst *Capsicum* accessions in the present study, and the values of which are quite comparable with that of different chilli accessions using different marker system [3, 59, 79]. Thus, the results of both the primer polymorphism and cross-transferability confirmed the extent of primer efficiency amongst chilli accessions through DNA fingerprinting process.

The techniques that measure the genetic polymorphism at genomic level are indispensable for identifying genetically and ecologically distinct populations and which can be used for the genetic improvement and breeding program in the desired populations. Therefore, the identification of genetic information such as *Na*, *Ne*, *I*, *He*, and *uHe* are very crucial for genetic characterization of populations using molecular markers. At species level, the increased level of genetic variation was observed within *Capsicum annumm* than in *Capsicum baccatum* and lowest was seen in *Capsicum frutesense.* A species with higher genetic variation owes it to its widespread ecological distribution, robust environmental adaptation, survivability, and evolutionary consequences [80]. Among the *Capsicum* population, the mean value for *Nei*’*s* gene diversity, *Ht*, and *Hs* were 0.376, 0.296, and 0.180, respectively, which is similar to previous reports of genetic structure analysis in populations involving other plants [81–83]. Such correlation studies using molecular markers have not been reported in *Capsicum* species, though correlation studies involving fruit characteristics with that of fruit diseases are reported [48]. Thus, a significant level of genetic variation was identified amongst different *Capsicum* accessions using ISSR markers and this genetic differentiation is influenced by several factors such as population size, reproduction patterns, cross-pollination or out crossing, genetic drift, and gene flow which are associated in the rise of genetic diversity within the population [84–86].

The coefficients of gene differentiation (*Gst*) and gene flow (*Nm*) are important indices for genetic differentiation within and across the population. *Gst* values are classified into low (*Gst*<0.05), median (0.05<*Gst*), and high (*Gst*>0.15) for genetic differentiation in the population [87]. Likewise, the values of *Nm* also varied from greater than 1 to less than 0.1 for determining the qualitative analysis for genetic differentiation within and across the population [88]. In present study, values of *Gst* and *Nm* were found to be 0.393 and 0.773, respectively, in *Capsicum* population, but such studies are yet not reported in chilli. However, several earlier reports have been documented on population genetics in other plant species such as [Gst (0.381) and *Nm* (0.835)] in *Dipteroniadyerana* surveyed by ISSR marker [89], and in another study on genetic structure of *Jatropha curcas* by microsatellite-based marker (ISSR and DAMD) system, *Gst* and *Nm* were reported to be 0.4053 and 0.8085, respectively [90].

In the present study, the increased *Gst* value indicates an enhanced genetic differentiation within the population but dropdown in the value of *Nm* represented low level of gene flow or allelic migration among the population due to genetic drift [80, 89] which indicates random fluctuations in the allelic frequency or gene variants in a population developed by chance over the time during evolution. Genetic mutations are responsible for creating allelic diversity and forces of such genetic drift and gene flow also add to genetic variations and are known to be an essential component in the framework of genetic diversity information. Mutation, drift and selection pressure make a dynamic balance in the amount of allelic diversity in the species that allow individuals to adapt into different environmental conditions. It is observed that a small size of the population is coupled to genetic drift which causes loss of rare alleles and decreases the gene pool which might play an influential role in the evolution of new species [91, 92]. The small population structure or absence of population structure often exhibit low genetic diversity due to genetically similar populations [93], common origin, restricted distribution of population, restricted gene flow, and homogenous reproduction [94]. Thus, the natural selection, genetic drift, and gene flow or allelic migrations are very important phenomenon that are coupled with the changes in the allele frequencies over time, and if population encountered one or more of these forces, it can result in the violation of Hardy-Weinberg assumptions, and evolution occurs [95].

The plasticity in the population structures was evaluated by AMOVA and Jaccard’s similarity coefficient, and the result of AMOVA represented a significant genetic variation within the population of *Capsicum* accessions with 89% of total variability and 11% among the population of chillies. Such analysis have been reported for *Glycyrrhiza uralensis* [96], *Parkia timoriana* [97], *Trachyspermum ammi* [98], *Melocanna baccifera* [69], and *Solanum* species [71]. The result of the ANOVA is in consistent with *Gst* and *Nm* values wherein increased genetic differentiation was observed within population and reduced gene flow among the population. This increased variation within different chilli accessions may be due to distinct ecological conditions, adaptations, and variations in morphological characteristics in chillies. Also, polymorphism of different microsatellite repeats offers a great efficacy to identify inter- and intra-specific genetic polymorphism [99].

According to Jaccard’s similarity coefficient and UPGMA clustering analysis, the varied level of relationships revealed with low, moderate, and extensive genetic association among the different *Capsicum* accessions. Wherein two major groups of associations were observed in the present study along with a few loosen connection. Alike pictorial representations of population structure were supported by Euclidean similarity index or correlation matrix with Fruchterman-Reingold algorithm, principal coordinate analysis (PCoA), and structure analysis which exhibited consistency of results in the characterization of *Capsicum* accessions. Due to varied genome size, morpho-physiological variation, and distinct agro-ecological environments, the result of the present study represented a significant genetic relationship among chilli accession. Important factors which explain these results regarding harmony and discordance among the chilli accessions are the nature of marker system used, level of polymorphism, the number of detected loci, and region coverage of genome by each marker [100], occurrence of distributions either local or geographically distinct spawning groups, natural selection as well as adaptation, survivability, and evolution in changing environments [101, 102].

Thus, the effect of each factor or combined effects of multiple factors have an impact on mechanism that shapes the population genetic structure while extensively related and dissimilar genetic variations might be associated with increased and reduced amount of genetic information respectively. Therefore, evaluation of genetic diversity is an important factor for explicating the connection among various chilli accessions which is essential component of germplasm characterization. Identification and characterization of new variations from the germplasm will help to develop new cultivars with improved agronomic trait, useful for crop improvement, and breeding program in chillies.

## Conclusion

The study reveals valuable information about genetic polymorphism, cross-transferability, and genetic and structural plasticity among 54 accessions of chilli using ISSR markers. A significant amplification profiles were obtained which reflects marked genetic polymorphism and cross-transferability among chilli accessions indicating efficiency of ISSR markers for genetic discrimination and conservation among chilli accessions. A significant level of genetic information was revealed by the estimation of various factors (*Na*, *Ne*, *I*, *uHe*, *Hs*, *Ht*, and Nei’s statistics) which highlighted the molecular variability among different chilli accessions. Thus, the knowledge obtained through genetic variability could be used in the management of chilli germplasms. High level of coefficient of gene differentiation (*Gst*) represented restricted gene flow (*Nm*) due to genetic drift which can be correlated with high or low rate of allelic acquisition, adaptation, mating nature, interaction with different ecological conditions, and changes in morphological distinctiveness during the course of evolution. The present study provides a fundamental insight for germplasm characterization, genetic arrangement, and population structure of chilli germplasm which could be utilized in the effective management and selection of superior germplasm for breeding purposes.

## Data Availability

No additional data is included in the manuscript.

## Abbreviations

ISSR: Inter-simple sequence repeats
PCR: Polymerase chain reaction
CTAB: Cetyl trimethyl ammonium bromide
PIC: Polymorphism information content
*I*: Shannon’s information index
*H*: Expected heterozygosity
DP: Discriminating power
RP: Resolving power
*GST*: Coefficient of gene differentiation
*Nm*: Gene flow
PCoA: Principal coordinate analysis (PCoA)
AMOVA: Analysis of molecular variance
UPGMA: Unweighted pair group method with arithmetic average
PAST: PAleontological statistics
